# Angiogenesis in Interstitial Lung Diseases: a pathogenetic hallmark or a bystander?

**DOI:** 10.1186/1465-9921-7-82

**Published:** 2006-05-25

**Authors:** Argyris Tzouvelekis, Stavros Anevlavis, Demosthenes Bouros

**Affiliations:** 1 Department of Pneumonology, Medical School, Democritus University of Thrace, Greece

## Abstract

The past ten years parallels have been drawn between the biology of cancer and pulmonary fibrosis. The unremitting recruitment and maintenance of the altered fibroblast phenotype with generation and proliferation of immortal myofibroblasts is reminiscent with the transformation of cancer cells. A hallmark of tumorigenesis is the production of new blood vessels to facilitate tumor growth and mediate organ-specific metastases. On the other hand several chronic fibroproliferative disorders including fibrotic lung diseases are associated with aberrant angiogenesis. Angiogenesis, the process of new blood vessel formation is under strict regulation determined by a dual, yet opposing balance of angiogenic and angiostatic factors that promote or inhibit neovascularization, respectively. While numerous studies have examined so far the interplay between aberrant vascular and matrix remodeling the relative role of angiogenesis in the initiation and/or progression of the fibrotic cascade still remains elusive and controversial. The current article reviews data concerning the pathogenetic role of angiogenesis in the most prevalent and studied members of ILD disease-group such as IIPs and sarcoidosis, presents some of the future perspectives and formulates questions for potential further research.

## Introduction

The interstitial lung diseases (ILDs) are a heterogeneous group of diffuse parenchymal lung diseases comprising different clinical and histopathological entities that have been broadly classified into several categories [1,2] including sarcoidosis and idiopathic interstitial pneumonias (IIPs). The latter have been recently classified into seven different disease-members [3-8]. The most important and frequent of these conditions are idiopathic pulmonary fibrosis (IPF) with the histopathologic pattern of usual interstitial pneumonia (UIP), non-specific interstitial pneumonia (NSIP) and cryptogenic organizing pneumonia (COP). Their aetiology has remained elusive and the molecular mechanisms driving their pathogenesis are poorly understood. Recent theories implicate recurrent injurious exposure, imbalance that shifts Th1/Th2 equilibrium towards Th2 immunity and angiogenesis in the pathogenesis of pulmonary fibrosis, both in human and experimental studies [9]. The Th1/Th2 pathway and angiogenesis have been recently suggested to play pivotal role in the immunopathogenesis of sarcoidosis contributing to the formation of granuloma, the main histopathologic feature of the disease [10].

The scope of this review article is to summarize the current state of knowledge regarding angiogenic and angiostatic activity in the most important and prevalent members of ILD disease-group such as IIPs and sarcoidosis, discuss its pathogenetic role and present some of the future perspectives and limitations based on authors' assessment or originated from the statements of original authors.

### 1. Definitions

Angiogenesis is the process of new capillary blood vessels growth and is instrumental under both physiologic and pathologic conditions. Physiologic conditions include embryogenesis, growth, tissue repair after injury and the female reproductive cycle whereas pathologic angiogenesis can occur in chronic inflammatory and fibroproliferative disorders and tumorigenesis of cancer. Angiogenesis is similar to but distinct from vasculogenesis which describes the *de novo *formation of blood vessels from angioblasts or endothelial progenitor cells, process that mostly occurs during embryogenesis [11]. On the other hand, angiogenesis describes the sprouting of new vessels from pre-existing vasculature which can occur both in embryonic and adult life. The regulation of angiogenesis is determined by a dual, yet opposing balance of angiogenic and angiostatic factors that promote or inhibit neovascularization, respectively.

### 2. Angiogenic mediators in interstitial lung diseases (Table 1)

**Table 1:** List of studied angiogenic and angiostatic mediators in ILDs

**Angiogenic mediators**
**CXC chemokines containing the ELR motif**
• GRO-a/CXCL1
• GRO-b/CXCL2
• GRO-γ/CXCL3
• ENA-78/CXCL5
• GCP-2/CXCL6
• NAP-2/CXCL7
• IL-8/CXCL8

**Growth Factors**
• VEGF
• bFGF

**Angiostatic mediators**

**CXC chemokines that lack the ELR motif**
• PF-4/CXCL4
• MIG/CXCL9
• IP-10/CXCL10
• ITAC/CXCL11
• CXCL14

**Growth Factors**
• PEDF
Abbreviations: bFGF: basic fibroblast growth factor, GCP: Granulocyte chemotactic protein, GRO: Growth related genes, IP-10:IFN-γ-inducible -protein 10, ITAC: IFN-γ-inducible T-cell a chemoattractant, MIG: Monocyte Induced by interferon gamma-protein, NAP: Neutrophil activating protein, PEDF: Pigment epithelium growth factor, PF: Platelet factor,, VEGF: Vascular growth factor

Molecules that originally promote angiogenesis include members of the CXC chemokine family, characteristically heparin binding proteins which on structural level have four highly conserved cysteine amino acid residues, with the first two cysteines separated by one nonconserved amino acid residue. CXC chemokines display unique diverse roles in the regulation of angiogenesis resulting from dissimilarity in structure. Therefore, members that contain in the NH_2_-terminus a three amino-acid motif (ELR) such as IL-8/CXCL8, epithelial neutrophil activating protein (ENA)-78/CXCL5, growth-related genes (GROs, a, β, γ/CXCL1, 2, 3), granulocyte chemotactic protein (GCP)-2/CXCL6 and neutrophil activating protein (NAP)-2/CXCL7, originally promote angiogenesis [11,12]. There are two candidate CXC chemokine receptors that mediate this effect: CXCR1 and CXCR2 [11,12]. Another crucial promoter of angiogenesis is vascular endothelial growth factor (VEGF) a dimorphic glycoprotein with multifunctional roles in both the development of vasculature and the maintenance of vascular structure and function [13,14]. Its expression is induced when most cell types are subjected to hypoxia [15]. Finally, another positive regulator of aberrant vascular remodeling in pulmonary fibrosis is basic fibroblast growth factor (bFGF) which has been shown originally to stimulate the proliferation of cells of mesodermal origin, including fibroblasts [16]. In addition, it has been shown that inappropriate expression of bFGF can result in tumor production through promotion of uncontrolled cell proliferation and aberrant angiogenesis [17-19].

### 3. Angiostatic mediators in interstitial lung diseases (Table 1)

By contrast, other members of the CXC chemokine family that do not contain the angiogenic ELR motif (ELR-) behave as potent inhibitors of angiogenesis. Platelet factor-4 (PF-4)/CXCL4 was the first chemokine described to inhibit aberrant angiogenesis. Furthermore, the angiostatic ELR- members of the CXC chemokine family include the interferon (IFN)-γ inducible protein (IP)-10/CXCL10, monokine induced by IFN-γ (MIG)-2 and IFN-γ-inducible T-cell a chemoattractant (ITAC)/CXCL11 [11,12]. The latter inhibit angiogenesis via interaction with the specific CXC chemokine receptor CXCR3 which is expressed in Th1 and natural killer (NK) cells. Additionally, pigment epithelium-derived factor (PEDF) is an inhibitor of new vessel formation, first described in retinal pigmented epithelial cells during diabetic retinopathy and then in young proliferating fibroblasts [20]. Its expression in retinal cell lines has been documented to be directly regulated by VEGF [21]. PEDF angiostatic activities are specific for new developing vessels and its expression has been detected in kidney, pancreas, prostate, pleura, testes, bone, within peripheral blood cells and recently in lung [21].

### 4. Pathogenetic pathways during aberrant angiogenesis

**Figure 1:**
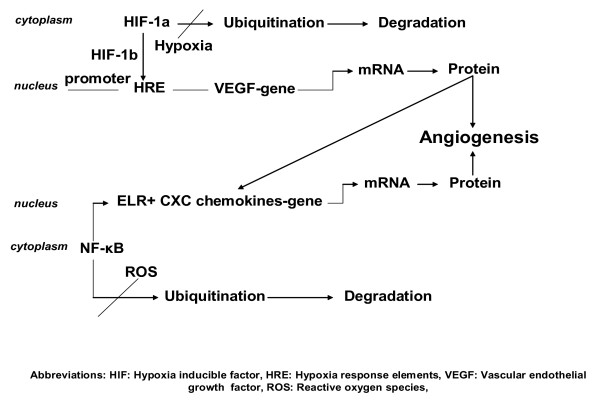
Schematic representation of the two major pathogenetic pathways regulating angiogenesis in pulmonary fibrosis. Under normal oxygen conditions HIF-1a is subject to ubiquitination and proteasomal degradation. Under hypoxic conditions, its ubiquitination is inhibited and HIF-1a is activated through the same kinase pathways with NF-κB and translocates to the nucleus. There it dimerizes with HIF-1b and the heterodimer recognizes specific allelic sequences located within the hypoxia response element found in the promoter region of several target genes (i.e VEGF). In addition, VEGF may directly promote the expression of angiogenic chemokines (i.e CXCL8) from endothelial cells in an autocrine and paracrine way. Generation of reactive oxygen species and activation of kinase pathogenetic pathways converges and activates NF-κB and sets in motion a process that releases NF-κB in the cytoplasm and leads to its translocation into the nucleus. There, all the promoters of angiogenic CXC chemokines contain a putative *cis*-element that recognizes and binds the transcriptional factor resulting to the activation of target genes and ultimately to protein synthesis.

Several transcription factors play instrumental role in promoting angiogenesis and sensing the environmental cues that drive this process. Strieter et al. [22] identified two transcription factors that stand out and appreciated the "master switches" that control aberrant angiogenesis. These are nuclear factor-κB (NF-κB) and hypoxia inducible factor-1a (HIF-1a). Both factors are under strict regulation. NF-κB plays an essential role as a "master switch" in the transactivation of angiogenic CXC chemokines as shown in detail for CXCL8 (Figure 1). Generation of reactive oxygen species activates NF-κB and sets in motion a process that releases NF-κB in the cytoplasm and leads to its translocation into the nucleus where it binds with the promoters of angiogenic CXC chemokines resulting to the activation of target genes [23]. In addition, it has been shown that VEGF promotes the expression of angiogenic chemokines (i.e CXCL8) from endothelial cells in an autocrine and paracrine way [13] (Figure 1). On the other hand, HIF-1a serves as a critical transcription factor for cellular and systemic oxygen homeostasis. Under hypoxic conditions HIF-1a is subsequent to activation and translocation into the nucleus. There it dimerizes with HIF-1b and the heterodimer recognizes the hypoxia response element found in the promoter region of several target genes (i.e VEGF) resulting to gene expression (Figure 1) [24,25].

## Angiogenesis in Interstitial Lung Diseases

### a. Angiogenesis in Idiopathic Interstitial Pneumonias (Tables 2, 3, 4)

**Table 2:** Human studies investigating angiogenic and angiostatic parameters in patients with idiopathic interstitial pneumonias (1997–2003)

**Investigator (year)**	**Tissue samples Sample size**	**IIP**	**Studied Parameters**	**Summary**	**Limitations**
Keane et al. ^41^(1997)	Lung specimens/50 patients/54 controls	IPF	CXCL8, 10	Increased levels of CXCL8,10 that favor angiogenesis	Incomplete analysis of the angiogenic network / In vivo micropocket assay
Lappi-Blanco et al.^53 ^(1999)	Lung specimens/19 patients	IPF-COP	VWF, CD34+		Small sample size / Lack of knowledge regarding factors responsible for vascular heterogeneity
Meyer et al. ^43 ^(2000)	BALF samples/32 patients/66 controls	IPF-CF-SARCO	VEGF	Decreased VEGF levels in IPF patients	Small number of patients / No correlation between serum and BALF levels / No correlation with clinical parameters of disease severity
Keane et al. ^42 ^(2001)	Lung specimens/91 patients/78 controls	IPF	CXCL5	Increased CXCL5 levels in IPF patients	Incomplete analysis of the angiogenic network
Lappi-Blanco et al. ^54^(2002)	Lung specimens/19 patients	IPF-COP	VEGF, bFGF	Increased VEGF and bFGF levels in MB compared to FF	Small sample size / Lack of knowledge regarding angiostatic regulators
Koyama et al. ^44 ^(2002)	BALF samples/49 patients/27controls	IPF-PF/CTD-SARCO	VEGF	Decreased VEGF levels in IPF patients	High variability between serum and BALF levels in health and disease
Renzoni et al. ^45 ^(2003)	Lung specimens/17 patients/12 controls	CFA-SSc	Vascular density and distribution	Abnormal vascular distribution in areas proximal to gas exchange / Phenotypically altered vessels	Morphometric study not suitable to identify the role of angiogenesis in hypoxemia

Abbreviations: BALF: Bronchoalveolar lavage fluid, bFGF: basic fibroblast growth factor, CF: Cystic fibrosis, CFA: Cryptogenic fibrosing alveolitis, COP: Cryptogenic organizing pneumonia, FF: Fibroblastic foci, IFN-γ: Interferon gamma, IIPs: Idiopathic Interstitial Pneumonias, IPF: Idiopathic pulmonary fibrosis, MB: Masson bodies, NSIP: Non-specific interstitial pneumonia, PF-CTD: Pulmonary fibrosis associated with a connective tissue disease, SARCO: Sarcoidosis, VEGF: Vascular endothelial growth factor

**Table 3:** Human studies investigating angiogenic and angiostatic parameters in patients with idiopathic interstitial pneumonias (2004–2005)

**Investigator (year)**	**Tissue samples Sample size**	**IIP**	**Studied Parameters**	**Summary**	**Limitations**
Ebina et al. ^48 ^(2004)	Lung specimens/7 patients/3 controls	IPF	Vascular density CD34+, VWF, CXCL8, VEGF	Heterogeneous increase in CD34+ alveolar capillaries / Morphologically altered vessels	Small sample size / Potential bias vascular density
Simler et al. ^56 ^(2004)	Serum samples/49 patients	IPF-NSIP-DIP	VEGF, CXCL8, ET1	Correlation of angiogenic cytokines with functional and radiological markers of disease severity	Heterogeneous group of IIPs / Patients not age and sex matched with controls / Lack of serial radiological data / Limited number of patients
Strieter et al. ^58 ^(2004)	BALF-serum samples/32 patients	IPF	CXCL11	Upregulation of CXCL11 levels in IPF patients after treatment with IFN-γ	No correlation with parameters of disease progression p values were not adjusted for multiplicity
Cosgrove et al. ^50 ^(2004)	Lung specimens/15 patients/12 controls	IPF-COP	PEDF-VEGF	Elevated PEDF and decreased VEGF levels within the FF. Increased VEGF levels within MB	In vitro angiogenic assay is less robust than the in vivo one / Small sample size
Nakayama et al. ^55 ^(2005)	BALF samples/27 patients/12 controls	IPF-NSIP	CXCL5, 10	Increased levels of CXCL5 and decreased levels of CXCL10 in patients with IPF compared to NSIP	Discrepancies between BALF and serological data / Limited number of patients
Belperio et al. ^52 ^(2005)	Lung specimens/BALF samples/68 patients/47 controls	BOS	CXCL1, 3, 5, 7, 8 CXCR2	Increased levels of CXCR2/CXCR2 ligands in lung biopsy and BALF samples from patients with BOS	Lack of evaluation of the angiostatic CXCR3/CXCR3 ligands axis
Pignatti et al. ^57 ^(2005)	BALF and serum samples/47 patients/10 controls	IPF-other ILDs	CXCR3, CCR4	Correlation of elevated CXCR3 levels with clinical parameters of disease severity in IPF patients	Lack of serial data in half of patients / No correlation with several parameters of disease severity / Discrepancies between serum and BALF levels

Abbreviations: BALF: Bronchoalveolar lavage fluid, COP: Cryptogenic organizing pneumonia, DIP: Desquamative Interstitial Pneumonia, FF: Fibroblastic foci, IFN-γ: Interferon gamma, IIPs: Idiopathic Interstitial Pneumonias, IPF: Idiopathic pulmonary fibrosis, MB: Masson bodies, NSIP: Non-specific interstitial pneumonia, PEDF: Pigment epithelial growth factor, VEGF: Vascular endothelial growth factor, VWF: Von Willebrand factor

**Table 4:** Studies investigating tissue angiogenic and angiostatic parameters in experimental models of pulmonary fibrosis

**Investigator (year)**	**Model**	**Studied Parameters**	**Summary**	**Limitations**
Keane et al. ^61^(1999)	BPF	MIP-2	Increased levels of MIP-2 in BPF mice / Inhibition of angiogenesis and fibrosis with neutralizing Abs	Model not representative of IPF
Keane et al. ^62^(1999)	BPF	CXCL10	Decreased CXCL10 levels / CXCL10 administration reduced BPF and angiogenic response	Model not representative of IPF
Jiang et al. ^65 ^(2004)	BPF	CXCR3	Regulation of BPF by CXCR3	Model not representative of IPF / Incomplete analysis of angiogenic network
Tager et al. ^78 ^(2004)	BPF	CXCL10	Inhibition of BPF by CXCL10	Model not representative of IPF / Incomplete analysis of angiogenic network
Burdick et al. ^63 ^(2005)	BPF	CXCL11	Systemic administration of CXCL11 inhibited BPF by altering aberrant vascular remodeling	Model not representative of IPF / Incomplete analysis of angiogenic network
Belperio et al. ^52 ^(2005)	Murine BOS	CXCL1, 2, 3 CXCR2, VEGF	Increased CXCR2/CXCR2 ligands' levels / Unchanged levels of VEGF / Neutralization of CXCR2 attenuated angiogenesis and BOS	Model has heterotopic positioning and discounts influence of adjacent airway mucosa
Hamada et al. ^64 ^(2005)	BPF	VEGF, sflt-1	Anti-VEGF gene therapy attenuates lung injury and fibrosis in BPF mice	Model not representative of IPF / Incomplete analysis of angiogenic network

Abbreviations: BPF: Bleomycin-induced pulmonary fibrosis, IPF: Idiopathic pulmonary fibrosis, VEGF: Vascular endothelial growth factor

The past ten years parallels have been drawn between the biology of cancer and pulmonary fibrosis. The unremitting recruitment and maintenance of the altered fibroblast phenotype with generation and proliferation of immortal myofibroblasts is reminiscent with the transformation of cancer cells [26-37]. A hallmark of tumorigenesis is the production of new blood vessels to facilitate tumor growth. A number of novel treatments targeting angiogenesis are in varying stages of clinical development for cancer [38]. On the other hand several chronic fibroproliferative disorders including IIPs are associated with aberrant angiogenesis [39]. In parallel with the biology of the fibroblast proliferation and deposition of ECM in IIPs, a considerable number of studies have examined the role of angiogenesis/vascular remodeling in wound healing and its contribution to the fibroproliferation and ECM deposition characterizing these disorders [39].

### i. Human studies (Tables 2 and 3)

There is increasing evidence supporting the notion that vascular remodeling in fibroproliferative disorders appears to be regulated by an imbalance between angiogenic and angiostatic factors. Seminal observation implicating angiogenic activity as an important aspect of progressive fibrosis was originally made by Turner-Warwick in 1963, when she demonstrated the presence of anastomoses between the systemic and pulmonary microvasculature in lungs of patients with IPF [40]. Despite these data suggesting a potential role of neovascularization in fibrogenesis, the exact contribution of aberrant vascular remodeling to the progression of fibrosis has been, so far, largely ignored. On the other hand, the pathology of IPF demonstrates temporal and regional heterogeneity and presents with distinct pathogenetic components compared to other IIPs that may explain major discrepancies in terms of clinical course, prognosis and responsiveness to treatment. On the basis of this conception, the last decade, a number of reports addressed intriguing questions arising from the above data. These include the following: 1) Is the primary vascular abnormality a lack or an excess of neovascularization and consequently what is the role of angiogenesis in the fibrotic process? 2) Is there any association of vascular remodeling with the histopathologic pattern of the IIP? or 3) any correlation with parameters of disease severity?

#### 1) Is there too much or too little?

Keane and colleagues were the first addressing this crucial issue. They demonstrated increased angiogenic activity in a large number of IPF lung specimens [41,42] and speculated that there it may be an opposing balance of angiogenic (CXCL8, CXCL5) and angiostatic factors (CXCL10) that favors angiogenesis [41,42]. However, other reports made the role of angiogenesis in IPF controversial. Meyer et al. [43] and Koyama et al. [44] documented depressed VEGF BALF levels in IPF patients compared to a variety of diffuse parenchymal lung diseases or healthy controls. However, an extremely high variability of serum and BALF VEGF levels in health and disease has been reported, which is provoked by numerous factors. These include epithelial cell apoptosis, cellular injury, proteolytic degradation due to smoking and aging [44].

Original attempt to prove an association between abnormal vasculature and regional heterogeneity characterizing IPF was performed by Renzoni and coworkers [45]. Fueled by previous studies showing marked decrease of interstitial vascularity in areas of extensive fibrosis [46,47], authors reported clusters of phenotypically altered vessels immediately adjacent to areas of active fibrosis in patients with two different forms of fibrosing alveolitis; IPF and fibrosing alveolitis associated with systemic sclerosis. One of the most intriguing aspects of this study was the demonstration of a substantial vascular redistribution leading to a great proportion of vessels removed from areas of gas exchange. This evidence was further corroborated by Ebina et al [48]. Authors effectively assessed by image analysis of dual immunostaining (CD34+, von Willebrand factor-VWF) the interstitial vascular density against the histologic severity of IPF. One of the most remarkable ascertainments of this study was the observation that both increased capillary density and vascular regression are found in the same disease, according to extent and severity of pulmonary fibrosis. Nevertheless, these findings instead of answering the original question generated novel hypotheses and gave birth to new dilemmas. What is the exact role of the increased angiogenic activity found in the least fibrotic areas? Is it involved in the fibrogenic process, is it a compensatory response or it prevents it? Authors hypothesize that the aberrant vascularity is compensatory to the vascular ablation seen in areas of extensive fibrosis and may be beneficial for the regeneration of the alveolar septa [49]. Nonetheless, further studies are warranted to support this concept. With this aim in mind, Cosgrove et al. [50] focused on the fibroproliferative areas of COP and UIP and reported, in agreement with previous reports [45,48], decreased vascular density within the fibroblastic foci. Scrutinizing for potent anti-angiogenic molecules, authors found for the first time a marked overexpression of a powerful angiostatic mediator, PEDF, within the fibroblastic foci but not in the Masson bodies. This finding was in contrary with prior studies [41,42], in which angiogenesis was promoted rather than suppressed. This disparity in the angiogenic activity can be explained by the use of different angiogenic assays or by the regional and temporal heterogeneity of IPF and can simply reflect pathological differences [51]. Finally, Belperio et al. [52] demonstrated aberrant vascular remodeling in lung specimens of patients with bronchiolitis obliterans pneumonia and corroborated this observation in BALF samples where they documented upregulated angiogenic activity.

#### 2) Is there any association of angiogenic activity with the histopathologic pattern of the IIP?

Lappi-Blanco et al. addressed this crucial issue [53,54]. They were the first who performed a comparative study on the net angiogenic activity found in two different forms of IIPs (UIP and COP) clinically and histologically distinguishable. A pronounced vascular remodeling in the fibromyxoid lesions of COP compared to the fibroblastic foci of UIP was reported [53]. In another study, same group of authors demonstrated a distinct expression of vascular growth factors (VEGF and bFGF) within the intraluminal connective tissue of UIP and COP [54]. Differential angiogenic profiles were also described by Cosgrove et al. [50] who demonstrated increased angiostatic activity within IPF lungs compared to COP tissue samples. In addition, Nakayama et al. [55] documented a local predominance of angiogenic factors (CXCL5) in IPF patients and angiostatic factors (CXCL10) in subjects with idiopathic NSIP.

#### 3) Is there any correlation with parameters of disease severity?

There is a great lack of knowledge regarding this issue which has been largely ignored. Renzoni et al. [45] were the first addressing this issue. They performed a morphometric analysis of the interstitial vascularity in two different types of fibrosing alveolitis and stated an inverse relation between alveolar-arterial oxygen gradient and the proportion of vessels close to areas of gas exchange, evidence that could explain the increased hypoxemia seen in these patients. However, as it pointed out by the authors, this study was morphometric and thus, unsuitable from its origin to evaluate markers of disease severity and correlate them with immunologic parameters.

Towards this direction, Simler et al. [56] performed a translational research of angiogenic cytokines (IL-8, VEGF, endothelin-1) and associated them with clinical parameters of disease progression over a 6-month period, in patients with IIPs. Patients with progressive lung disease demonstrated higher plasma levels of all three cytokines than non-progressors according to functional and clinical criteria. In addition, a positive relationship between the change in HRCT fibrosis score and the change in plasma VEGF and a negative relationship between the percentage change in forced vital capacity and the change in plasma VEGF was noted. Potential limitations include the analysis of a heterogeneous group of diseases, enrolment of a limited number of subjects, not age and sex matched with the controls and lack of serial radiological data. However, investigators performed the first longitudinal study in this field and identified potential prognosticators of disease progressiveness, an area that has severely hindered clinical research in ILDs.

A second attempt to correlate local and systemic expression of angiogenic mediators with clinical biomarkers of disease severity and activity was recently published by Pignatti et al [57]. They investigated the role of CXCR3 compared to CCR4 known to mediate Th2 response and reported a predominance of a Th2 microenvironment in IPF patients. An imbalance of the CXCR3/CCR4 expression in BALF T lymphocytes was well correlated with functional and radiological parameters of disease severity, speculating that these immunomodulators could function as prognostic guides of the disease course.

Finally, Strieter et al. [58] recently published the only, so far, study supporting the notion that mortality in IPF patients could be potentially improved through the anti-angiogenic properties of IFN-γ 1b supporting its therapeutic utility. More prospective studies in well defined subgroups of IIPs are needed to strengthen this assertion and assess the clinical utility of biomarkers of disease activity [59].

### ii. Experimental models (Table 4)

The vascular remodeling phenomenon has been also described in the experimental model of bleomycin-induced pulmonary fibrosis. The role of neovascularization during the pathogenesis of experimental pulmonary fibrosis was originally raised by Peao and coworkers [60]. In line with human data [40] investigators reported aberrant vascular remodeling in the peribronchial areas of the lungs proximal to fibrotic regions and accompanied by architectural distortions of the alveolar capillaries. While these eloquent studies implicated the presence of angiogenesis in the pathogenetic cascade of IPF, so far, there have been no investigations to delineate factors that regulate neovascularization and subsequent fibrosis. To demonstrate proof of the principle that CXC chemokines regulate angiogenic and angiostatic activity in IPF, Keane et al. effectively assessed the relevance of macrophage inflammatory protein [61] and CXCL10 [62] with the augmented net angiogenic activity in the in vivo model of pulmonary fibrosis. Neutralization of (MIP)-2 attenuated both angiogenic activity and the fibrotic response to bleomycin, whereas a relative deficiency of IFN-γ inducible angiostatic regulator CXCL10 was also noted. In addition, systemic administration of CXCL10 inhibited fibroplasia and angiogenesis, supporting the premise that aberrant angiogenesis enhances fibroblast proliferation and ECM deposition. In agreement with these findings, Burdick et al. [63] stated that instillation of the angiostatic CXCL11 produced a marked decrease of fibrotic areas and an attenuation of the dysregulated vascular remodeling.

Fueled by the prospect that anti-angiogenic treatment could be beneficial for pulmonary fibrosis, Hamada et al. [64] tested the efficacy of anti-VEGF gene therapy in the bleomycin model of pulmonary fibrosis. Administration of a specific VEGF receptor that blocks its activity produced a significant anti-fibrotic, anti-inflammatory and anti-angiogenic effect, suggesting an important role for VEGF through its versatile properties. In addition, Jiang et al. [65] used CXCR3 deficient mice and delineated potential mechanisms through which the CXCR3/CXCR3-ligands biological axis exerts a protective role by shifting the Th equilibrium toward resolution of the injurious response. Moreover, Belperio et al. [52] by using a murine model of bronchiolitis obliterans syndrome (BOS) conducted a proof-of-concept analysis and demonstrated that multiple angiogenic CXC chemokines and their receptors (CXCR2) are involved in a dual fashion in the pathogenetic pathway of experimental BOS.

The latter results have clear therapeutic implications since inhibition of angiogenic mediators or administration of angiostatic chemokines reduced lung collagen deposition and attenuated the exaggerated matrix remodeling. On the basis of this concept, neutralization of proangiogenic environment should be pursued. However, the latter statement should be treated with caution for the following reasons: 1) Findings derived from the bleomycin model of pulmonary fibrosis may not be applicable to human disease since pathogenetic components seen in bleomycin-induced pulmonary fibrosis do not demonstrate areas compatible with fibroblastic foci, the leading edge of human fibrosis. In addition, there are clear limitations to this model in terms of its self-limiting nature, the rapidity of its development and the close association with inflammation that accompanies the lung injury [66]. Regarding the experimental model of BOS, it also presents with substantial weaknesses due to its heterotopic positioning, discounting the influence of adjacent airway mucosa [52]. 2) Moreover, the aforementioned studies were unable to investigate the complete angiogenic and angiostatic network involved in the pathogenesis of the disease. Therefore, results could be misleading due to the lack of knowledge of a variety of mediators that may have a direct effect on fibroblast proliferation and collagen gene expression. Therefore, their potential contribution to vascular and matrix remodeling can not be excluded. Maybe an investigation of several angiogenic pathways in a single experiment could help us circumvent this problem. However, the above limitations are not to diminish the scientific value and accuracy of these studies but to underline the necessity for further analyses using more representative experimental models in combination with human studies.

#### b. Angiogenesis in sarcoidosis (Table 5)

**Table 5:** Studies investigating angiogenic and angiostatic parameters in patients with sarcoidosis

**Investigator (year)**	**Tissue samples Sample size**	**Studied Parameters**	**Summary**	**Limitations**
Agostini et al. ^69 ^(1998)	Lung specimens/BALF samples/24 patients/6 controls	CXCL10	Increased expression of CXCL10 in sarcoid tissues / Positive relation of elevated CXCL10 BALF levels with T cell alveolitis	Lack of knowledge regarding regulators of CXCL10 expression / Incomplete analysis of the Th1 response / Small sample size
Miotto et al. ^70 ^(2001)	Lung specimens/BALF/ 39 patients/10 controls	CXCL10, MCPs, eotaxin	Increased expression of CXCL10 levels in sarcoidosis patients	Expression of CXCL10 not selective for Th1 mediated response / Lack of association with parameters of disease severity
Sekiya et al. ^72 ^(2003)	Serum samples/33 patients	VEGF	VEGF as a prognosticator of disease activity and extent	Retrospective analysis No serial measurement / No relation with serological parameters of disease severity / Limited number of patients
Katoh et al. ^71 ^(2005)	BALF and serum samples	CXCL9, 10	Increased BALF concentrations in sarcoidosis patients	Discrepancies between BALF and serum levels / No relation with clinical parameters of disease severity

Abbreviations: BALF: Bronchoalveolar lavage fluid, MCPs: Monocyte chemotactic proteins, VEGF: Vascular endothelial growth factor

Recent immunological advances on sarcoidosis have revealed a T helper 1 (Th1) and T helper 2 (Th2) paradigm with predominance of the Th1 response in its immunopathogenesis [67,68]. The last years have seen the emergence of Th1 mediators with pleiotropic properties including the IFN-γ-regulated CXC chemokines that lack the ELR motif (ELR-) at the NH_2 _terminus. While CXCR3/CXCR3 ligands inhibit angiogenesis, CXCR3 ligands play a pivotal role in orchestrating Th1 cytokine-induced cell-mediated immunity via the recruitment of mononuclear and CD4+ T-cells expressing CXCR3 and consequently via the granuloma formation (1). So far, there are only few studies in the literature implicating angiogenesis in the immunomodulatory cascade of sarcoidosis and correlating its immunopathogenesis with members of the angiostatic group of CXC chemokines. These studies are discussed in the following lines.

The concept of disparate activity of the IFN-γ-induced CXC chemokines in the context of Th1-like immune disorders, such as sarcoidosis, was originally raised by Agostini et al. [69] who documented an enhanced expression of IP-10 in sarcoid tissues and a positive relationship of BALF IP-10 levels and the degree of T-cell alveolitis, suggesting its pivotal role in ruling the migration of T-cells to sites of ongoing inflammation. In addition, Miotto et al. [70] described a specific for Th1 mediated response upregulation of IP-10 BALF levels further implicating angiostatic CXC chemokines in the inflammatory cascade of sarcoidosis. Recently, Katoh et al. [71] reported elevated BALF concentrations of IP-10 and MIG in patients with sarcoidosis and chronic eosinophilic pneumonia. Furthermore, Sekiya et al. [72] demonstrated a strong correlation of elevated VEGF serum levels with clinical parameters of disease activity and severity in sarcoidosis patients indicating a potential usefulness as a predictor of disease extent and responsiveness to treatment.

The aforementioned studies substantiate the assertion that IFN-γ-induced CXC chemokines are strongly involved in the immunomodulatory cascade of sarcoidosis implicating angiostasis with Th1 immune response. However, there are several arguments that should be addressed. The majority of the studies cited above have investigated the ability of CXCR3 ligands to promote Th1-dependent immunity and not to inhibit angiogenesis. Studies have shown that angiostatic CXC chemokines are more likely to contribute to the granuloma formation through their chemotactic rather than angiostatic properties. The contention of "immunoangiostasis" (promotion of Th1 response and at the same time inhibition of angiogenesis) as it has been coined out by Strieter et al. [11] may possibly support the infectious aetiology of sarcoidosis suggesting that the hypovascular central area of the sarcoid granuloma can contain the microbe in a dormant sate and at the same time promote its eradication through Th1 mediating factors and the recruitment of T cells. Therefore, it is tempting to speculate that factors that regulate angiogenesis and promote aberrant vascular remodeling can shift the Th1/Th2 equilibrium to Th2 immune response resulting to fibrotic sarcoid phenotypes associated with detrimental prognosis and clinical course. However, there is major lack of knowledge regarding this issue. Future analyses of the angiogenic microenvironment in well defined subgroups of patients with sarcoidosis with and without pulmonary fibrosis are warranted to elucidate the role of angiogenesis during this pathogenetic process and support this concept.

## Future challenges and limitations

IIPs are a heterogeneous group of diffuse parenchymal disorders resulting from damage to the lung parenchyma by varying patterns of inflammation and fibrosis. On the other hand several patients with sarcoidosis develop irreversible lung damage and pulmonary fibrosis which culminates to a fatal outcome. Several theories and mechanisms have been delineated regarding the pathogenesis of fibrotic lung disorders. Recent evidence support the concept that inflammation is subsequent to injury and that fibrosis occurs as a polarization of the Th2 immune response of the body to repeated injury to the lung ("multiple hits" hypothesis) [9,73].

**Figure 2:**
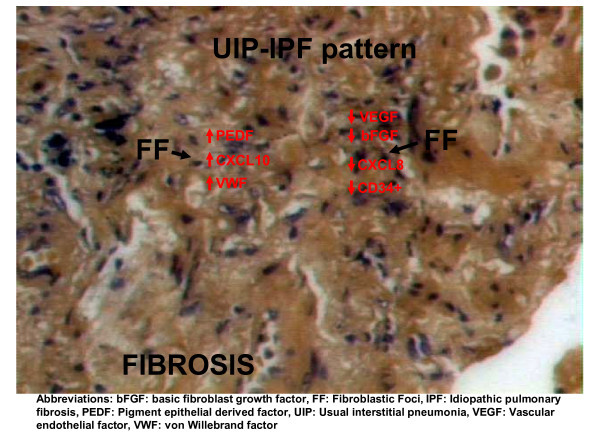
Expression of angiogenic and angiostatic mediators within the fibroblastic foci in UIP-IPF pattern. Red arrows demonstrate the increased or decreased expression of angiogenic and angiostatic regulators within areas of active fibrosis.

Putting the aforementioned data together, we tentatively present the three current theories regarding the role of aberrant vascular remodeling in the fibrogenic process. The first hypothesis is based on the idea that the hypervascularity observed in the least fibrotic areas has a role in the regeneration of the alveolar septa damaged by the fibrotic process and is a compensatory response to the decreased vascularity seen within the fibroblastic foci (Figure 2). In this case, the primary deficiency is the inability to form new vessels in areas of extensive fibrosis and consequently inhibition of angiogenesis could be detrimental [31]. Therefore the vascular ablation in areas proximal to gas exchange may lead to an increased distance to be travelled by oxygen and provide a plausible mechanism of the striking hypoxemia seen in end stage disease [28]. This is an interesting theory; however, there is lack of evidence to substantiate it. A comparative study of the HIF-1a-VEGF axis in different areas of the same disease process or in different histopathologic patterns could be a possible approach to this crucial issue. Potential disruption of this pathway can explain the inability of lung to respond to various stresses and injuries by the induction of VEGF resulting to reduced endothelial and epithelial cell viability that characterizes pulmonary fibrosis.

Alternative hypothesis regarding the role of vascular remodeling during the process of ECM remodeling has also emerged. This theory supports the premise that newly formed microvessels enhance the exaggerated and dysregulated ECM deposition, support fibroproliferation and inhibit normal epithelial repair mechanisms [50]. Human [51,52] and animal [61-64] data has shown that inhibition of angiogenic mediators is followed by a significant attenuation of the fibrotic process. Therefore it is tempting to speculate that the increased angiogenic activity observed in lung biopsies from patients with pulmonary fibrosis facilitates the progression and expansion of the fibrotic lesions in a similar way that promotes tumor growth and metastasis [74]. Turner-Warwick et al. [40] originally demonstrated that the vascular supply of the fibrotic regions derives from the systemic circulation through systemic-pulmonary anastomoses. This observation correlates with the recently emerged theory of circulating fibrocytes according to which bone marrow-derived cells behave like mesenchymal stem cells and extravasate into sites of tissue injury and contribute to pulmonary fibrosis [9,75-77]. Hence, angiogenic cytokines in parallel with their chemotactic properties may facilitate migration of fibroblasts at areas of tissue injury by formation of new blood vessels which may help to provide fibrotic regions with the nutrient supplies needed for cellular proliferation and differentiation. However, findings from current studies [50,53,54] question this hypothesis on the basis of the striking hypovascularity within the areas of active fibrosis. Nevertheless, the natural history of IIPs and especially IPF includes a series of overlapping events and is characterized by a temporal and regional heterogeneity [9]. Thereby, the finding of vascular heterogeneity is compatible and logical and supports the concept that angiogenesis is a major or minor contributor of the fibrotic process depending on the stage and the severity of the disease course. A longitudinal angiogenic study of biopsy specimens from patients with fibrotic lung disease of different histopathologic patterns is crucial to elucidate the role of vascular remodeling during different time points of the disease course.

The third theory supports the notion that the role of angiogenesis in the pathologic process of pulmonary fibrosis is overestimated and that aberrant vascular remodeling is just a bystander or a consequence of fibrogenesis. The latter idea is based on the assertion that CXC chemokines may exert their anti-fibrotic activities through pathogenetic pathways different from those of angiogenesis [78]. The aforementioned observation coupled with major controversies regarding the sequential pathologic events culminating to pulmonary fibrosis give credence to the view that angiogenesis is just a bystander or a consequence of the fibrogenic process and is not actively involved in its initiation and progression. Although, authors are not strong supporters of this theory, however it should not be excluded.

Based on the above data we can state that although several study groups have investigated aberrant vascular remodeling in the pathogenesis of pulmonary fibrosis, the relative roles played by new vessel formation and vascular regression in IPF and subsequently in other fibrotic lung disorders are still elusive and controversial. However, to address this issue further investigation in the context of large prospective multicenter studies using highly standardized techniques is sorely needed. The emergence of massive genome screening tools (DNA microarrays) [79] coupled with reliable validation techniques (tissue microarrays) [80] can help scientists to illuminate the interplay between vascular and matrix remodeling in the pathogenesis of fibrotic ILDs and elevate its current state of knowledge to the same level as for angiogenesis in tumor growth and metastasis.

## Conclusion

Several lines of research have been proven inadequate to demystify the relative role of angiogenesis in the etiopathogenesis of chronic fibroproliferative disorders. The question originally raised still remains unanswered: "A pathogenetic hallmark of just a bystander?" However, the status of knowledge regarding the contribution of newly formed vessels in the initiation and/or progression of the sequential events of abnormal injurious response, paradoxical apoptosis and exaggerated matrix remodeling has been greatly elevated by several studies. So far, a number of investigations give credence to the view that a chemokine imbalance favoring angiogenesis supports fibroproliferation and inhibits normal repair mechanisms. Alternatively, the regional vascular heterogeneity in IPF can be explained as a compensatory response (vascular regression) to the striking hypovascularity described in areas of active fibrosis. Currently, angiogenesis represents one of the most fruitful applications in the therapeutic minefield of fibrotic lung disorders. The lack of an effective treatment option challenges chest physicians to think beyond conventional therapeutic strategies and apply fresh approaches. Blockage of multiple angiogenic mediators may provide a way forward. Whether our hopes will be fulfilled or disproved remains to be seen.

## Abbreviations

Basic fibroblast growth factor (bFGF)

Bronchiolitis obliterans-organizing pneumonia (BOOP)

Bronchiolitis obliterans syndrome (BOS)

Cryptogenic organizing pneumonia (COP)

Epithelial neutrophil activating protein (ENA)-78

Extracellular matrix (ECM)

Farmer's lung disease (FLD)

Granulocyte chemotactic protein (GCP)

Growth-related genes (GROs)

Hypoxia inducible factor-1a (HIF-1a)

High resolution computed tomography (HRCT)

Idiopathic pulmonary fibrosis (IPF)

Interferon-γ (IFN-γ)

(IFN)-γ inducible protein (IP)-10

(IFN)-γ inducible T cell-a chemoattractant (ITAC)

Idiopathic interstitial pneumonias (IIPs)

Interstitial lung diseases (ILDs)

Matrix metalloproteinases (MMPs)

Macrophage inflammatory protein (MIP)-2

Monokine induced by IFN-γ (MIG)-2

Natural killer (NK) cells

Neutrophil activating protein (NAP)-2

Nuclear factor-κB (NF-κB)

Non-specific interstitial pneumonia (NSIP)

Platelet factor-4 (PF-4)

Pigment epithelium-derived factor (PEDF)

Systemic sclerosis (SSc)

Usual interstitial pneumonia (UIP)

Vascular endothelial growth factor (VEGF)

von Willebrand factor (VWF)

## Competing interests

The author(s) declare that they have no competing interests.

## Authors' contributions

AT and DB were involved with the study conception. AT and SA performed the data acquisition and interpretation. AT prepared the manuscript. DB was involved in revising the article for important intellectual content. All authors read and approved the final manuscript.
